# BlaSTorage: a fast package to parse, manage and store BLAST results

**DOI:** 10.1186/1751-0473-8-4

**Published:** 2013-01-30

**Authors:** Massimiliano Orsini, Simone Carcangiu

**Affiliations:** 1 CRS4, Bioinformatics Group, Loc Pixina Manna, Pula 09010, ITALY

**Keywords:** BLAST, Blast parser, Python-package, Serialized python object

## Abstract

**Background:**

Large-scale sequence studies requiring BLAST-based analysis produce huge amounts of data to be parsed. BLAST parsers are available, but they are often missing some important features, such as keeping all information from the raw BLAST output, allowing direct access to single results, and performing logical operations over them.

**Findings:**

We implemented BlaSTorage, a Python package that parses multi BLAST results and returns them in a purpose-built object-database format. Unlike other BLAST parsers, BlaSTorage retains and stores all parts of BLAST results, including alignments, without loss of information; a complete *API* allows access to all the data components.

**Conclusions:**

BlaSTorage shows comparable speed of more basic parser written in compiled languages as C++ and can be easily integrated into web applications or software pipelines.

## Findings

Today, it is quite common in computational biology to be working with large sequence datasets. An operation that often needs to be performed is the search by similarity. The tools for these similarity-based searches are often based on BLAST-core [1,2], except for software specialized for short reads. The output of BLAST is based on sets of pairwise alignments between query and reference sequence(s), along with some metadata about the alignments such as *e-value, similarity score, query name, etc*. Running with multiple inputs, BLAST produces a results file for each sequence queried or, alternatively, a unique multi-result file. As the input increases in number and size of queries, manual inspection of BLAST results quickly becomes an impractical procedure. The problem is accentuated when results have to be assessed, compared, or passed to other software tools. BLAST parsers can then be used to automatically filter results by some criteria or to eliminate unneeded fields from records (for example: *subject title, similarity, identity*). To overcome the limits of simply storing results in flat files, they could be placed in a relational database, but that approach adds significant complexity to the system.

Parsers can be roughly divided into three main analysis style: i) by *Model* (Pull parsing), ii) by *Events* (Push parsing), iii) hybrid. Pull parsers work reading the entire document creating an internal representation of the whole document. On the other hand, Push parsers read the data incrementally. Hybrid parsers represent a compromise between the two other approaches, loading only a block of the document at a time and leaving it up to the application code to decide when to ask for another block. None of these approaches is generally better than the others; rather, each is better suited for different types problems.

There exist several well-known, stand alone BLAST parsers: BioPerl [3], MuSeqBox [4], Zerg [5] and its Perl/Python wrappers [6], Boulder [7]. They are hybrid parsers, more closely resembling the Pull approach since in case of multi-BLAST results they need to iterate over the entire document to access to extract data.

We present a new BLAST parser called BlaSTorage. Its development was motivated by the need to interpret a very large amount of BLAST results without losing any part of the data they contain, and the need to query the BLAST result sets. The BlaSTorage parsing algorithm was developed paying close attention to the computation time it requires. It could be considered a hybrid parser, since it reads over the BLAST result stream like a Push parser during the reading and storing phase. Then, once the results have been completely scanned it generates a model of the entire result set, similarly to Pull parsers. The BlaSTorage engine extracts the various sections of each result by applying a number of simple regular expressions. It then converts each section’s data to a *serialized python object* and stores all these to a single special database file. A complete *API* allows the user to access to every part of this database to query it (a simple example is given in the next paragraph). Moreover, the *API* throws exceptions to help the application manage incomplete or corrupted results and generate useful error messages. The BlaSTorage module is available at the following URL: http://biowiki.crs4.it/biowiki/MassimilianoOrsini

To evaluate BlaSTorage’s performance we compared it to other publicly available stand-alone parsers (Table 1). We measured the time taken by each program to parse three blastp results files of growing sizes (see Table 1 legend). BlaSTorage resulted about two orders of magnitude faster than other parsers that retain alignments and allow database-like querying, such as Boulder and BioPerl. BlaSTorage was about one order of magnitude faster, at least with large files, than MuSeqBox, which is written in C++, and it was slightly faster than Zerg::Report, a Perl module for BLAST reports; it is important to note that none of these latter parsers retain alignments. BlaSTorage showed slightly lower speed than Zerg-Perl and Pyzerg, two interfaces to Zerg C libraries written in Perl and Python respectively [6], and obviously was slower than the Zerg-C parser [5] which is implemented in C. Yet, unlike BlaSTorage, none of the mentioned Zerg-based parsers are able to retain alignment information or access a specific result of a multi-blast output.

**Table 1:** BlaSTorage comparative performances

**Parser (language)**	**DB**	**Alignm. retain**	12.3mb *	180mb *	1.8gb *
boulder (perl)	yes	yes	213 ± 1.5	-	-
Bio::SearchIO (perl)	no	yes	27 ± 0.3	1495 ± 12	148506
MuSeqBox (C++)	no	no	0.73 ± 0.04	552.1 ± 1.5	-
Zerg::Report	no	no	1.47 ± 0.12	122.3 ± 0.8	-
**Blastorage (python)**	**yes**	**yes**	**1.4 ± 0.8**	**37.9 ± 0.13**	**490 ± 12**
zerg (perl)	no	no	0.36 ± 0.01	26.7 ± 0.16	341 ± 6
Pyzerg	no	no	0.35 ± 0.03	24.43 ± 0.3	312 ± 13
Zerg C *)blastp output, STD over five replicates	no	no	0.08 ± 0.0	2.01 ± 0.04	74 ± 7

In this table are shown results of some of the most popular stand-alone BLAST parsers. The time taken (seconds) to parse different blastp output files was measured in five separate runs using a laptop with 2 Gb RAM, 2 CPU, 1.83 Ghz and an intel centrino processor. Missing values are referred to tests where the program crashed or was terminated after 24 hours of unproductive work (extensive tests in supplementary material).

The design of BlaSTorage offers some advantages over many other available parsers. First, it allows one to retain the alignment part of the BLAST results, which is usually discarded. Second, it can be easily included in pipelines and web applications by using methods in the *api.py* and *manage.py* modules (the latter contains methods to export parsed/filtered results toward the standard output or to a file, see Additional file 1 for some examples). Third, the storage object structure, together with *api* and *manage* classes, implement a database-like access to the results, with the possibility of applying logical operations (the manage class contains methods to filter, select and sort results using an SQL-like syntax; see Additional file 1 supplementary material for some examples). Finally, once the BlaSTorage database is built and written to disk, the user can access it directly, without re-parsing the BLAST output file; this approach results in great time savings. The ability of BlaSTorage to scale with large files is highlighted by our performance tests (Additional file 1 ), It has proven to be a helpful tool when alignments have to be analyzed or when results have to be accessed in random order. In applications where these features are not important other faster tools should be considered. One limitation of the current version of BlaSTorage is that it is not compatible with all BLAST versions; it currently works well with versions up to and including 2.2.26+. The current release works with the blastp, blastn, blastx and psiblast programs, and we are working to extend it to also tblastx and tblastn.

Two graphical BLAST parsers have to be cited for completeness, but have been excluded from our tests since their features are not directly comparable. The first of these, Batch Blast Extractor [8], has a user-friendly GUI to present information from BLAST output and can produce a tab-delimited text which can be used for downstream analysis. However, it does not return the alignments. The second, NOBLAST-JAMBLAST [9], is more of a results manager than a feature-complete parser. Nevertheless, it shows a plethora of features including some statistical treatment of data. It uses the new tabular output of BLAST (-m 18/19, not present in older releases) that contains the alignments, and together with the JAMBLAST program it provides a complete graphical interface to filter and manage BLAST results. Alas, NOBLAST requires a MySQL database to be installed and working knowledge of SQL to perform logical operations on results. For these reasons we did not consider these two packages as competitors of BlaSTorage, since we believe that they solve different problems. In our opinion, graphical applications are useful when handling a small quantities of data or as tools for users who are not comfortable with programming. Although BlaSTorage can be used by command line in a relatively intuitive way through its *API*, its principal design goal was for it to be easily included into pipelines or web applications. For example, in our laboratory BlaSTorage is systematically used to evaluate contigs obtained by *de novo* assembly of RNA-seq data. In this kind of application assembled contigs are blasted against well-known reference transcriptomes generating output files of about 1-1.5 GB in size. BlaSTorage has also been used in the Pariga server (http://resources.bioinformatica.crs4.it/pariga/, unpublished), a web application that performs all-against-all BLAST searches given two sequence datasets, mainly designed for ortholog discovery. In this particular implementation of BlaSTorage has been optimized by storing data with *PyObjCTools* rather than the standard *shelve* libraries. This change results in improved performance at the cost of the inconvenience of having to write a db-storage file for each input sequence. For the standard version of BlaSTorage we decided this inconvenience was not acceptable, since multiple output files can generate confusion and add complexity, especially with large jobs.

BlaSTorage is optimized for speed and scalability in order to be able to manage large amounts of results without loss of information. A simple *API* allows accessing the database at run-time or any later time.

### A short interactive example

>>> from blastorage import Storage

>>> dbFile = 'blast.db' # database filename

>>> inFile = 'blastResTest.blast'# blast results file name

>>> st = Storage(dbFile, inFile)

>>> st.store()

>>> api = st()

>>> print api.getGeneralInfo()

Database: uniref100

Posted date: May 30, 2007 11:40 PM

....... (continue)

>>> for blast in api: #One BlastResult for each query

… print blast.getQueryTitle()…

tgp_o21.Contig7

tgp_o21.Contig32

....... (continue)

>>> for blast in api:

… for iteration in blast: # multiple results in case of psiblast

… for align in iteration: #One Alignment for each subject

… print align.getSubjectTitle()…

UniRef100_Q2IMJ3

UniRef100_Q2IIH7

....... (continue)

>>> for blast in api:

… for iteration in blast: # multiple results in case of psiblast

… for align in iteration:

… for hsp in align: # One Hsp for each align

… print hsp.getGradesAsString()…

Score = 166 bits (419), Expect = 3e-39

Identities = 168/468 (35%), Positives = 184/468 (39%), Gaps = 69/468 (14%)

Score = 157 bits (397), Expect = 1e-36

Identities = 165/474 (34%), Positives = 180/474 (37%), Gaps = 79/474 (16%)

....... (continue)

>>> st.close()

#Once you closed the storage it can be accessed later:

>>> st = Storage('blast.db')

>>> api = st()

>>> etc…

#Filtering and Exporting Data using manage package

>>> from manage import Manager

>>> M = Manager(api)

>>> M.selectBlastResWhereEvalueLowerThan(0.001, header=True)

##query | iter | subject | e-value

ENSMUSP00000000095 0 UniRef100_E4Y6N2 1e-85

ENSMUSP00000000095 0 UniRef100_Q6J4Z5 1e-85

… (continue)

ENSMUSP00000000095 0 UniRef100_G0WKX4 2e-81

ENSMUSP00000000095 0 UniRef100_Q90WR1 1e-80

… (continue)

retrieved 2500 results; query time: 2.09233503342 seconds >>>

>>> M.selectBlastresWhereIdentityPercentageHigherThan(80, header=True)

##query | iter | subject | Ident.%

ENSMUSP00000000095 0 UniRef100_H9G432 95.0

ENSMUSP00000000095 0 UniRef100_Q70WD3 91.0

ENSMUSP00000000095 0 UniRef100_E0VMZ8 80.0

ENSMUSP00000000095 0 UniRef100_B1V8P8 90.0

ENSMUSP00000000095 0 UniRef100_B7PT63 88.0

… (continue)

ENSMUSP00000000095 0 UniRef100_Q535V1 89.0

ENSMUSP00000000095 0 UniRef100_Q90WR1 97.0

retrieved 853 results; query time: 1.72032017708 seconds

>>>

>>> M.sortBlastresByIdentityPercentage()

##query | iter | subject | Ident.%

ENSMUSP00000000145 0 UniRef100_Q5F2C5 100.0

ENSMUSP00000000145 0 UniRef100_Q8R1X1 89.0

ENSMUSP00000000145 0 UniRef100_Q91WC3 89.0

ENSMUSP00000000145 0 UniRef100_Q5ICG6 89.0

ENSMUSP00000000145 0 UniRef100_A8IP90 89.0

… (continue)

ENSMUSP00000000145 0 UniRef100_Q9UKU0-2 81.0

ENSMUSP00000000145 0 UniRef100_F7GMD5 81.0

ENSMUSP00000000001 0 UniRef100_Q9DC51 100.0

ENSMUSP00000000001 0 UniRef100_P08753 99.0

ENSMUSP00000000001 0 UniRef100_Q3TJH1 99.0

… (continue)

ENSMUSP00000000001 0 UniRef100_B5X3R5 89.0

ENSMUSP00000000001 0 UniRef100_Q4SAC0 82.0

ENSMUSP00000000096 0 UniRef100_P70325 100.0

ENSMUSP00000000096 0 UniRef100_D4A0A2 97.0

.. (continue)

ENSMUSP00000000028 0 UniRef100_E1FWQ3 23.0

retrieved 2500 results; query time: 1.08432006836 seconds

# direct access to a given result

>>> M.selectBlastresWhereQueryIdEqualTo('ENSMUSP00000000145', header=True)

##queryid len descr round subjectid eval align ident. Pos. gaps

ENSMUSP00000000145 622 0 UniRef100_Q5F2C5 0.0 622 100.0 100.0 0

ENSMUSP00000000145 622 0 UniRef100_Q8R1X1 0.0 622 89.0 89.0 75

ENSMUSP00000000145 622 0 UniRef100_Q91WC3 0.0 622 89.0 89.0 75

ENSMUSP00000000145 622 0 UniRef100_Q5ICG6 0.0 621 89.0 89.0 75

ENSMUSP00000000145 622 0 UniRef100_A8IP90 0.0 621 89.0 89.0 75

ENSMUSP00000000145 622 0 UniRef100_Q6IU14 0.0 604 87.0 87.0 75

ENSMUSP00000000145 622 0 UniRef100_H2M5V7 0.0 349 55.0 72.0 45

ENSMUSP00000000145 622 0 UniRef100_H3CZQ8 0.0 345 55.0 73.0 43

ENSMUSP00000000145 622 0 UniRef100_H3CZQ7 0.0 344 55.0 73.0 43

ENSMUSP00000000145 622 0 UniRef100_D3ZW20 0.0 351 56.0 73.0 45

ENSMUSP00000000145 622 0 UniRef100_H2SDA4 0.0 351 55.0 71.0 57

retrieved 251 results; query time: 0.2691218853 seconds

>>> M.selectBlastresWhereSubjectIdEqualTo('UniRef100_G5BKD1', header=True)

##queryid round subjectid eval align ident. Pos. gaps

ENSMUSP00000000145 0 UniRef100_G5BKD1 0.0 362 58.0 75.0 41

retrieved 1 results; query time: 0.097626886368 seconds

## Competing interests

The authors declare that they have no competing interests.

## Authors’ contributions

MO conceived of the project, wrote and refined some Python classes and drafted the manuscript. SC developed the software and performed the tests. All authors have read and approved the final manuscript.

## Supplementary Material

Additional file 1 BlaSTorage Manual. Click here for file
