# Supplementary material for: BlaSTorage: a fast package to parse, manage and store BLAST results
Source: Source Code Biol Med. 2013 Jan 30;8:4. doi: 10.1186/1751-0473-8-4 (PMC3571973; doi:10.1186/1751-0473-8-4)
Supplement: Additional file 1 — BlaSTorage Manual. [file 1751-0473-8-4-S1.pdf]

BlaSTorage Manual

July 2012

The BlaSTorage is a Python package that parses BLAST output files and returns results in a database-object format. It retains and stores all parts of BLAST results including alignments, without loss of information.

This parser/storage works with blast (blastn, blastp, blastx, psiblast) files in Pairwise Alignment format as returned by using BLAST with default parameters corresponding to the blast command `-outfmt 0` or `-m 0` in previous BLAST versions.

BlaSTorage works by converting BLAST results into a database-like Python object, where each section (see Figure 1) of the results file is accessible via a dedicated *API* (see below). The resulting model is built by streaming through the BLAST output file, while the BlaSTorage engine extracts each output section by using a set of simple regular expressions and converts each datum into a serialized Python object.

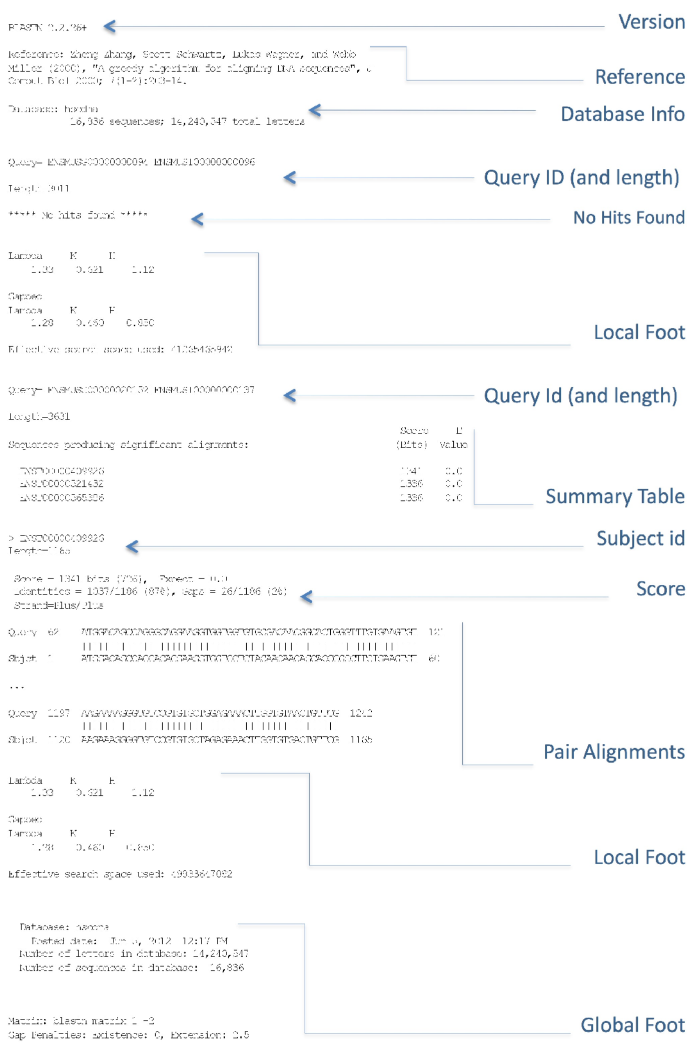

Figure 1: main sections of a BLAST results file imported into BlaSTorage. In the case of psiblast output, some of these sections are replicated for each iteration round.

BlaSTorage object structure
As previously mentioned, the BLAST results file is converted into an object with a nested structure, where sections of the output file are converted into an object format (Fig. 2).

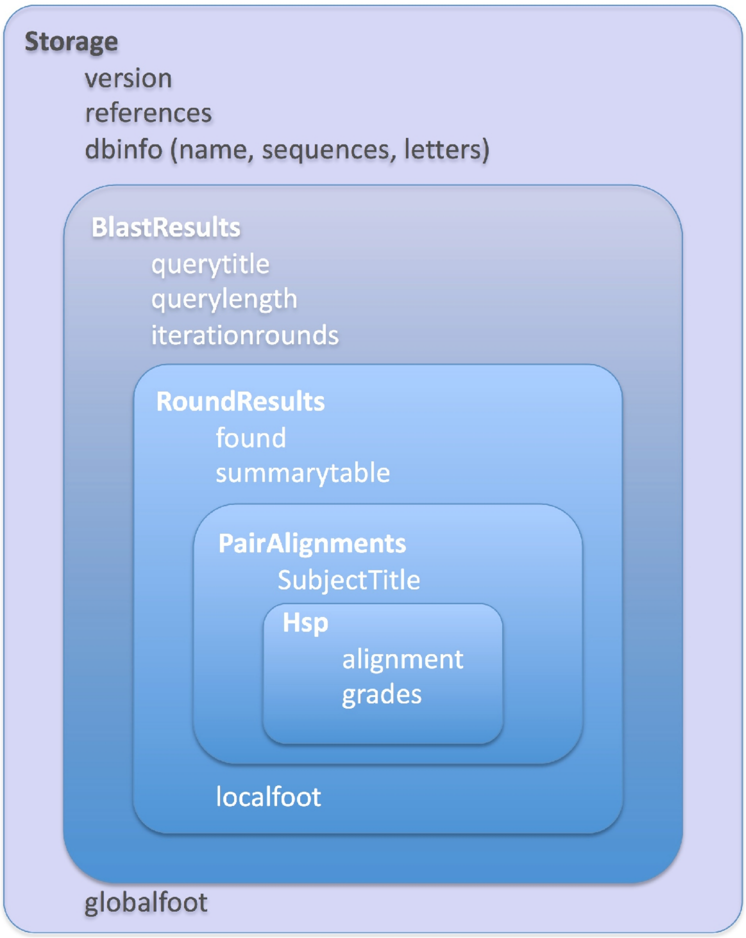

Figure 2: BlaSTorage Blast Model.

In the following table a brief description of BlaSTorage elements:

element	type	parent	note
**Storage**	**object**	-	the main object
version	attribute/string	Storage	Blast version as it appears in the file header
reference	attribute/string	Storage	References, as they appear in the file header
dbinfo	attribute/string	Storage	database name, sequences, letters
globalfoot	attribute/string	Storage	database info, matrix, gap penalties, etc.
**BlastResults**	**object**	Storage	object, for each query sequence
querytitle	attribute/string	BlastResults	query name, including length
querylen	attribute/string	BlastResults	query length as string
rounds	attribute/list	BlastResults	number of iteration for psiblast, 0 for other application
**RoundResults**	**object**	BlastResults	object, blast result for each (psi)blast iteration
found	attribute/boolean	RoundResults	boolean, false in case of “****No Hits found ****”
summarytable	attribute/list	RoundResults	summary table for each blast (iteration)
localfoot	attribute/string	RoundResults	Lambda, K, H parameters
**PairAlign**	**object**	RoundResults	object, pair wise alignment
subjectid	attribute/string	PairAlign	subject id, name and description
**Hsp**	**object**	PairAlign	object, single pairwise sequence match
grades	attribute/string	Hsp	alignment attibutes, identity, similarity, score, e-val, gaps
subjectalign	attribute/string	Hsp	query-subject sequence alignment

Installing blastorage BlaSTorage package

(sudo privileges required)

```
tar zxvf blastorage.tar.gz
cd blastorage
/your/python/interpreter/path/python setup install
```

you should see a message similar to:

```
running install
running build
running build_py
copying ./api.py -> build/lib.linux-x86_64-2.7/blastorage
copying ./scribe.py -> build/lib.linux-x86_64-2.7/blastorage
copying ./storage.py -> build/lib.linux-x86_64-2.7/blastorage
copying ./tests/backtesting.py -> build/lib.linux-x86_64-2.7/blastorage/tests
copying ./tests/test.py -> build/lib.linux-x86_64-2.7/blastorage/tests
running install_lib
copying build/lib.linux-x86_64-2.7/blastorage/storage.py -> /usr/local/lib/py-
thon2.7/dist-packages/blastorage
copying build/lib.linux-x86_64-2.7/blastorage/scribe.py -> /usr/local/lib/py-
thon2.7/dist-packages/blastorage
byte-compiling /usr/local/lib/python2.7/dist-packages/blastorage/storage.py to
storage.pyc
byte-compiling /usr/local/lib/python2.7/dist-packages/blastorage/scribe.py to
scribe.pyc
running install_egg_info
Removing /usr/local/lib/python2.7/dist-packages/blastorage-1.2.6.egg-info
Writing /usr/local/lib/python2.7/dist-packages/blastorage-1.2.6.egg-info
```

A short interactive example

```
>>> from blastorage import Storage
>>> dbPath = 'blast.db'
>>> blasteresPath = 'test.blastx'
>>> st = Storage(dbPath, blasteresPath)
>>> st.store()
>>> api = st()

>>> print api.getBlastVersion()
BLASTX 2.2.15 [Oct-15-2006]

>>> print api.getBlastReference()
Reference: Altschul, Stephen F., Thomas L. Madden, Alejandro A. Schaffer,
Jinghui Zhang, Zheng Zhang, Webb Miller, and David J. Lipman (1997),
"Gapped BLAST and PSI-BLAST: a new generation of protein database search
programs",  Nucleic Acids Res. 25:3389-3402.

>>> print api.getGeneralInfo()
  Database: uniref100
    Posted date:  May 30, 2007 11:40 PM
...........................................................................
...........................................................................
```

```
Neighboring words threshold: 12
Window for multiple hits: 40
X1: 16 ( 7.3 bits)
X2: 5 ( 1.9 bits)
X3: 64 (24.7 bits)
S1: 41 (21.7 bits)
S2: 28 (15.4 bits)


>>> print api.getQueriesNumber()
17
>>> print len(api)
17

>>> for blast in api:           # One BlastResult object for each query
...     for iteration in blast: # multiple results in case of psiblast
...         print blast.getQueryTitle()
...
tgp_o21.Contig7
tgp_o21.Contig32
.........................................................................
.........................................................................

>>> for blast in api:
...     for iteration in blast:     # multiple results in case of psiblast
...         for align in iteration: # One Alignment object for each subject with
                                    #almost one HSP (High-scoring Segment Pairs)
...             print align.getSubjectTitle()
...
UniRef100_Q2IMJ3
UniRef100_Q2IIH7
.........................................................................
.........................................................................

>>> for blast in api:
...     for iteration in blast:     # multiple results in case of psiblast
...         for align in iteration:
...             for hsp in align: # One Hsp object for each found High-scoring
                                  # Segment Pairs
...                     print hsp.getGradesAsString()
...
 Score =  166 bits (419), Expect = 3e-39
 Identities = 168/468 (35%), Positives = 184/468 (39%), Gaps = 69/468 (14%)
 Frame = -1

 Score =  157 bits (397), Expect = 1e-36
 Identities = 165/474 (34%), Positives = 180/474 (37%), Gaps = 79/474 (16%)
 Frame = -3

 Score =  152 bits (383), Expect = 5e-35
 Identities = 171/463 (36%), Positives = 187/463 (40%), Gaps = 78/463 (16%)
 Frame = -2
.........................................................................
.........................................................................

>>> st.close()
```

After you have closed the storage, if you want to access it again later you can instantiate a Storage object passing only the database path:

```
>> st = Store('blast.db')
>> api = st()
>> etc...
```

Testing blastorage package

A test script is included in the blastorage package. It first imports the input BLAST results (or a folder containing several BLAST files) into a blastorage-db and then rebuilds the original file(s) using the API classes. Finally, it compares (line by line) the original BLAST results file with the rebuilt copy. If the original and the rewritten files are identical it reports a successful message (including the time spent to parse, store e rewrite the results file); otherwise it reports the first incongruent line and the execution is halted.

```
cd blastorage/tests
/your/python/interpreter/path/python tests.py --dir=/path/containing/only/blast/files
```

otherwise

```
/your/python/interpreter/path/python test.py --path=/path/containing/one/file/blast
```

If successful you should see a message similar to:

```
$ python test.py --path=/path/containing/one/file/test.blast
File : /path/containing/one/file/test.blast
Test completed successfully!
Completed in: 0.187865018845 sec
```

API modules

The BlaSTorage API allows the user to access all parts of BLAST results. Some methods have been implemented simply to access parameters (e.g.. sequence identity, mismatches), other methods allow the user to iterate over BLAST results or return results in some desired order. These features could be helpful to integrate BlaSTorage in pipelines.

```
class Storage

methods:

getQueriesNumber(): Returns the queries number as int.

getBlastVersion(): Returns the blast Version e.g : BLASTP 2.2.15 [Oct-15-2006] as string.

getDatabaseInfo(): Returns info about database searched as string e.g. :
       Database: tesdb
           17,259,763 sequences; 6,123,185,472 total letters

getBlastReference(): Returns the reference(s) in the output header.
        e.g:
        Reference: Altschul, Stephen F., Thomas L. Madden, Alejandro A. Schaffer,
        Jinghui Zhang, Zheng Zhang, Webb Miller, and David J. Lipman (1997),
```

```
        "Gapped BLAST and PSI-BLAST: a new generation of protein database search
        programs",  Nucleic Acids Res. 25:3389-3402.
```

getGeneralInfo(): Returns info in the global foot as string:

```
       e.g.:
          Database: Uniprot
          Posted date:  Jun 3, 2007 10:06 PM
          Number of letters in database: 24,021,888
          Number of sequences in database:  47,776

        Lambda     K      H
           0.326    0.140    0.468

        Gapped
        Lambda     K      H
           0.267   0.0410    0.140

        Matrix: BLOSUM62
        Gap Penalties: Existence: 11, Extension: 1
        Number of Sequences: 47776
        Number of Hits to DB: 23,009,686
        Number of extensions: 1004485
        Number of successful extensions: 2647
        Number of sequences better than 10.0: 12
        Number of HSP's gapped: 2647
        Number of HSP's successfully gapped: 12
        Length of query: 444
        Length of database: 24,021,888
        Length adjustment: 107
        Effective length of query: 337
        Effective length of database: 18,909,856
        Effective search space: 6372621472
        Effective search space used: 6372621472
        Neighboring words threshold: 11
        Window for multiple hits: 40
        X1: 15 ( 7.1 bits)
        X2: 5 ( 1.9 bits)
        X3: 64 (24.7 bits)
        S1: 40 (21.7 bits)
        S2: 64 (29.3 bits)
```

getQueryTitles(): Returns a list of all the queries titles.

getBlastResult(queryid): Returns blast results of a given sequence by its queryid as BlastResult object

class BlastResult
methods:

getIterationRounds(): returns a list of iteration rounds in case of psiblast program, [‘0’] in case of other algorithms (blastp, blastn, blastx).

getQueryTitle(): Returns the query id as string

getQueryLength(): Returns query length as int

getQueryDescription(): Returns query description as string

getRoundResult(iterationround): Returns round results (table, alignments) of a given iteration round, a RoundResult object

class RoundResult
methods:

getSubjectsTitles(): returns subjectids of matched sequences as list

foundHits(): returns the presence of matching sequences as boolean

getSubjectsTable(): returns the summary table as string

getIterationRound(): returns the iteration round as int

getLocalInfo(): returns parameters of the search (Lambda, gamma, etc) of the pointed iteration round.

getAlignment(subjectid): returns query-subject alignment for a given subject id as Alignment object

class Alignment(object)

methods:
getSubjectTitle(): Returns subject id as string

getSubjectDescription(): return additional subject information if present in the subject fasta header

getSubjectLength(): return length of subject sequence as int

Alignment is an iterable object that returns Hsp objects

class Hsp(object)

methods:

setGradesDict(): set hsp-alignment parameters in a dictionary, it has to be called before accessing to alignments parameters

getGradesAsString(): returns hsp-alignment parameters as string
```
        e.g.:
         'Score =  258 bits (130), Expect = 6e-66
          Identities = 370/450 (82%)
          Strand = Plus / Plus'
```

getGradesAsDict(): Returns hsp-alignment parameters as a dict like:
```
          'Score': '242 bits (122)', 'Identities': '436/538 (81%)',
          'Expect': '4e-61', 'Strand': 'Plus / Plus', 'Gaps': '2/538 (0%)'
```

getScoreExpression(): Returns score as a string like: '22.3 bits (11)'

getScoreAsFloat(): Returns score as float, from the previous example it returns: 22.3

getIdentitiesExpression(): Returns identity as a string like: '11/11 (100%)'

getIdentitiesAsPercentage(): Returns a float as 100.0 from identity expression like: '11/11 (100%)'

getIdentitiesAsAbsoluteValue(): returns the number of identical matches as int from the expression : '11/11 (100%)'

getGapsExpression(): Returns identity as a string like: '2/538 (0%)' or None if there are no gaps

getGapsAsPercentage(): Returns gaps percentage as float

getGapsAsAbsoluteValue(): Returns total gaps as int

getPositivesExpression(): Returns a string '2/538 (0%)' or None if there are no positives (blastn)

getPositivesAsPercentage(): Returns positives percentage as float or None if there are no positives (blastn)

getPositivesAsAbsoluteValue(): Returns positives matches as int or None if there is no positives (blastn)

```
getEvalueAsFloat(): Returns evalue as float

getFrameExpression(): return 'Frame' as string from blastx jobs

getPairwiseAlignment(): returns alignments a a string like:
        "
        Query: 13  MVRGQVFDVGPRYTNLSYIGEGAYGMVCSAYDNVNKVRVAIKKISPFEHQTYCQRTLREI 72
                   M R   FD+  +Y  +  IGEGAYG VCSA    + ++VAIKKI PF  + +  RT+REI
        Sbjct: 1   MARTITFDIPSQYKLVDLIGEGAYGTVCSAIHKPSGIKVAIKKIQPFSKKLFVTRTIREI 60

        Query: 73  KILLRFR-HENIIGINDIIRAPTIEQMKDVYIVQDLMETDLYKLLKTQH-----LSNDHI 126
                   K+L  F  HENII I D +R  +I+++  VY+V++LMETDL K++  Q+     LS+DH+
        Sbjct: 61  KLLRYFHEHENIISILDKVRPVSIDKLNAVYLVEELMETDLQKVINNQNSGFSTLSDDHV 120 "
```

Including BlaSTorage into pipelines

Methods in manage.py package allow the user to access the BlaSTorage object using a db-like syntax. Three main groups of methods have been implemented: Select (they write to standard output results satisfying a given condition in a tabular format); Export (they act as select methods but they write results to a file, whose name has to be specified together with the desired output format: pairwise, as the original output, or tabular); Sort (this group of methods orders the results, in tabular format, according to a given criteria, as e-value, or identity, positives, etc.).

```
class Manager

methods:
# select group
selectBlastresWhereEvalueLowerThan(evalue, retmode):
       returns blastresults where e-value is lower than indicated, retmode: 'tabular'
(tab separated), 'pairwise' (as in the blast output)

selectBlastresWhereIdentityPercentageHigherThan(identity, retmode):
       returns blastresults where identity percentage is higher than indicated, retmode:
'tabular' (tab separated), 'pairwise' (as in the blast output)

selectBlastresWhereIdentityPercentageEqualTo(identity, retmode):
       returns blastresults where identity percentage is equal to the indicated value,
retmode: 'tabular' (tab separated), 'pairwise' (as in the blast output)

selectBlastresWherePositivePercentageHigherThan(positives, retmode):
       returns blastresults where positive percentage is higher than indicated, retmode:
'tabular' (tab separated), 'pairwise' (as in the blast output)

selectBlastresWherePositivePercentageEqualTo(positives, retmode):
       returns blastresults where poositive percentage is equal to the indicated value,
retmode: 'tabular' (tab separated), 'pairwise' (as in the blast output)

selectBlastresWhereGapsLowerThan(gaps, retmode):
       returns blastresults where gaps are lower than indicated, retmode: 'tabular' (tab
separated), 'pairwise' (as in the blast output)

selectBlastresWhereFrameEqualTo(frame, retmode):
       returns blastresults where Frame is equal to the indicated value, retmode: 'tabu-
lar' (tab separated), 'pairwise' (as in the blast output)

selectBlastresWhereCoverageHigherThan(coverage, retmode):
       returns blastresults where Coverage (calculated as: (aligned-gaps)/ query length)
is higher than indicated, retmode: 'tabular' (tab separated), 'pairwise' (as in the blast
output)


# sort group
```

```
# the following methods return results sorted as indicated in the name of the method it-
self, only tabular return mode has been implemented

SortBlastresByEvalue()
SortBlastresByIdentityPercentage()
SortBlastresByPositivePercentage()
SortBlastresByCoveragePercentage()


# export group
# they work as "select" methods, output filename has to be specified

SelectBlastresWhereEvalueLowerThan(evalue, retmode, filename)
SelectBlastresWhereIdentityPercentageHigherThan(identity, retmode, filename)
SelectBlastresWhereIdentityPercentageEqualTo(identity, retmode, filename)
SelectBlastresWherePositivePercentageHigherThan(positives, retmode, filename)
SelectBlastresWherePositivePercentageEqualTo(positives, retmode, filename)
SelectBlastresWhereGapsLowerThan(gaps, retmode, filename)
SelectBlastresWhereFrameEqualTo(frame, retmode, filename)
SelectBlastresWhereCoverageHigherThan(coverage, retmode, filename)
```

To work in automatic pipelines BlaSTorage methods have to be embedded into simpleb python scripts. Let us suppose you want to run a blast search, the you want to select only those blast results where Identity is higher than 85%. Write a script like this:

```
#! /your/python/interpreter
import sys
from blastorage import Storage
from manage import Manager
infile = sys.argv[1]                            #the name of the blast results file
db = infile.rsplit(".", 1)[0]+".db"             #suppose the db file has the same prefix
st = Storage(db, infile)                        #instance the Storage class
st.store()
api = st()                                            #instance the api class
M = Manager(api)                                      #instance the manager class
M.selectBlastresWhereIdentityPercentageHigherThan(85) #call the desired method
```

save your script, then change its permission:

```
you$ chmod +x yourscript.py
```

run your script

```
you$ ./yourscript.py blast.results.file
```

alternatively, if you want to reuse your script, the desired threshold (in this case the iden-tity) can be passed as external parameter:

```
#! /your/python/interpreter
import sys
from blastorage import Storage
from manage import Manager
infile = sys.argv[1]                            #the name of the blast results file
db = infile.rsplit(".", 1)[0]+".db"             #suppose the db file has the same prefix
identity = sys.argv[2]                          #the desired threshold
st = Storage(db, infile)                        #instance the Storage class
st.store()
api = st()                                                  #instance the api class
M = Manager(api)                                            #instance the manager class
M.selectBlastresWhereIdentityPercentageHigherThan(identity) #call the desired method
```

save your script, then change its permission:

```
you$ chmod +x yourscript.py
```

run your script
```
you$ ./yourscript.py blast.results.file 85
```

In this case the script can be easily reused simply passing the name of the file and the desired parameter. You can write a collection of scripts for generic use simply by using the manger class. In case of more complex parsing or filtering it is probably better to use basic methods in the api class. As example, let us suppose you want filter from your job blast results in order to obtain those queries retrieving at least five sequences matching queries with evalue lower than 0.001, identity higher than 85% and not more than 3 gaps:

```
from blastorage import Storage

db = 'your.db.file'
infile = 'your.blast.results.file'

st = Storage(db, infile)
st.store()
api = st()

desiredresults = {}
for blast in api:                           # iteration over queries
    desiredresults[blast.title]= []         # temporary dictionary
    for iteration in blast:                 # iteration over round in case of psiblast
        for alignment in iteration:         # iteration over alignments
            for hsp in alignment:           # iteration over hsps
                hsp.setGradesDict()         # convert hsp parameters in a dictionary
                evalue   = hsp.getEvalueAsFloat()
                identity = hsp.getIdentitiesAsPercentage()
                gaps     = hsp.getGapsAsAbsoluteValue()
                if evalue < 0.001 and identity >= 85 and gaps <= 3:# satisfying condition
                    desiredresults[blast.title].append(alignment.getSubjectTitle(), /
                                                       evalue, identity, gaps)

    if len(desiredresults[blast.title]) < 5:  # if not more than 5 results
        del desideredresults[blast.title]     # removing from temporary dictionary

print desideredresults.keys()                 # printing queries satisfying requirements
```

Performance

We report the results of a some of the tests we performed to evaluate the performance of BlaSTorage in terms of computation time and disk space usage for the generated db file.

Reference Databases: *nt* (nucleotides), *nr* (proteins)

Query Datasets: 10, 100, 1000, 10000, sequences randomly extracted from:
mouse transcriptome (Ensembl 67, protein coding genes, cdna sequences)
mouse proteome (Ensembl 67, protein coding genes, peptides sequences)

blastp, blastn, blastx, psiblast run with default parameters, psiblast run with 3 iterations.

In the following table are indicated both disk space occupancy and parsing performances.

For each dataset is reported the disk occupancy of the Fasta file, the occupancy of blast results file (according to the blast algorithm used) and the occupancy of the corresponding BaSTorage db file.

Moreover, for each dataset has been indicated the average time to parse the blast results file and write the db on the disk, followed by the time required to access the db after it was built. Standard deviation was calculated over five replicates performed on a AMD quad-core (2.9 Ghz), 8GB RAM. Results ordered by blast results disk occupancy.

**Fasta file size**	**inputed seq**	**Blast program**	**Blast results file size**	**BlaSTorage Occupancy**	**Parsing File Storing DB** (first access)	**Reading DB** (second access)
5.6 KB	Nt 10seq	blastx	1.8 MB	2.0 MB	0.15 ± 0.00	0.02 ± 0.00
5.6 KB	Nt 10seq	blastn	3.2 MB	3.4 MB	0.19 ± 0.02	0.02 ± 0.02
2.4 KB	Pr 10seq	blastp	5.0 MB	5.4 MB	0.32 ± 0.01	0.04 ± 0.01
2.4 KB	Pr 10seq	psiblast	19.8 MB	21.2 MB	1.22 ± 0.02	0.17 ± 0.02
156 KB	Nt 100seq	blastn	28.3 MB	29.7 MB	1.46 ± 0.01	0.21 ± 0.00
156 KB	Nt 100seq	blastx	44.1 MB	48.0 MB	3.20 ± 0.03	0.47 ± 0.02
57 KB	Pr 100seq	blastp	46.7 MB	50.4 MB	3.08 ± 0.07	0.44 ± 0.02
57 KB	Pr 100seq	psiblast	204.3 MB	217.6 MB	12.84 ± 0.07	1.78 ± 0.02
1.6 MB	Nt 1000seq	blastn	328 MB	345 MB	18.37 ± 0.18	2.69 ± 0.08
565 KB	Pr 1000seq	blastp	468 MB	508 MB	35.80 ± 1.13	6.57 ± 0.17
1.6 MB	Nt 1000seq	blastx	500 MB	544 MB	37.74 ± 0.59	7.16 ± 0.09
565 KB	Pr 1000seq	psiblast	1.9 GB	2.1 GB	84.98 ± 1.20	31.2 ± 0.19

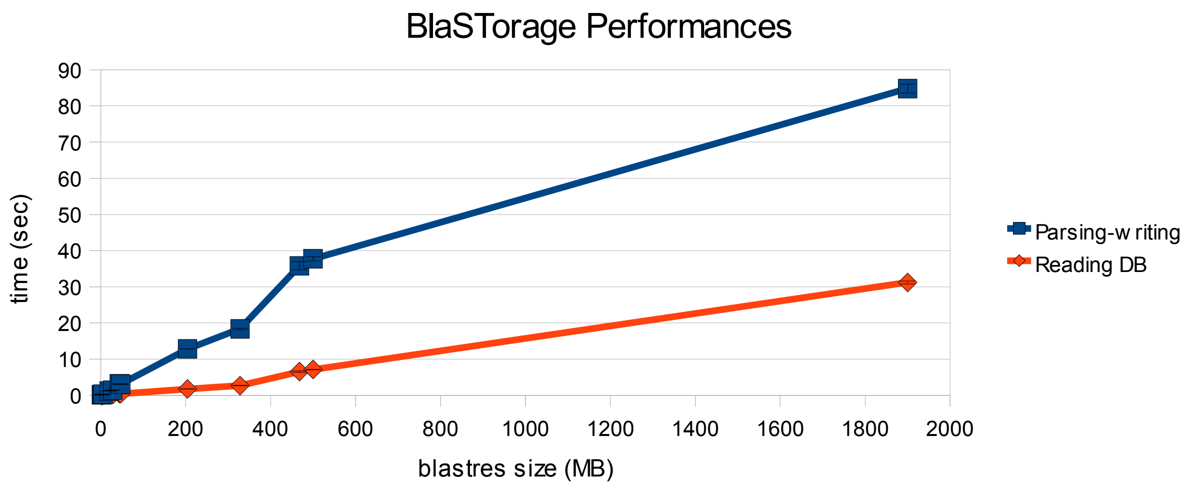

Limits

BlaSTorage is based on Python *shelve* libraries, when database is closed shelve library performs a synchronization of the db on the disk. This results in a large memory requirement with a consequent computational time increasing. This effect is appreciable with input file larger than 2 GB and it appears as a delay after the computation is terminated, Sometimes an “Out of Memory” error can occur even if the parsing have been correctly completed; unfortunately this provokes a not correct writing of the db on the disk.
Datasets in the following table have been correctly parsed with a 8GB RAM workstation, but failed to correctly write back the database on the di, the larger dataset (*) failed to write the db on the disk. **All of them can be normally parsed and queried (including results exporting) by an interactive python shell.**

Fasta file size	inputed seq	Blast program	Blast esults file size	BlaSTorage Occupancy	Parsing File Storing DB
565 KB	Pr 1000seq	psiblast	1.9 GB	2.1 GB	84.98 ± 1.20
14.2 MB	Nt 10000seq	blastn	3.7 GB	3.9 GB	147 ± 8
4.6 MB	Pr 10000seq	blastp	4.1 GB	4.3 GB	199 ± 11
14.2 MB	Nt 10000seq	blastx	5.0 GB	5.2 GB	250 ± 18
4.6 MB	Pr 10000seq	psiblast	17.4 GB	*	1235 ± 35

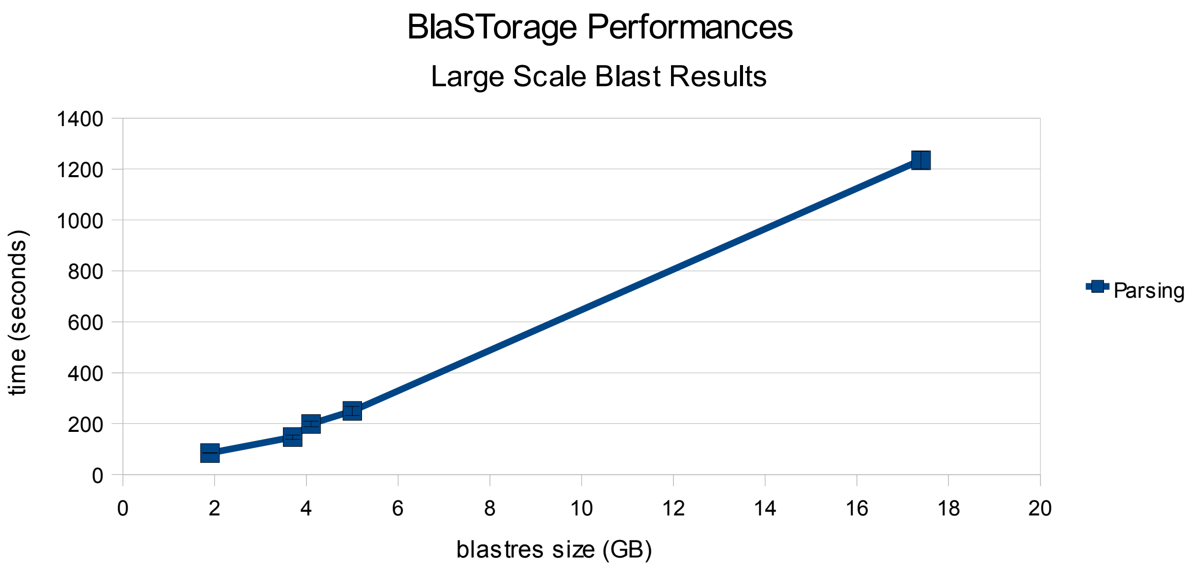

Future Development

In the future we are firstly planning to extend BlaSTorage to other programmes as tblastx, tblastn, and probably to derive a specific code for psiblast output. This would increase code readability and improve memory usage for those application that do not perform blast iterations (blastn, blastp, blastx). Then we are studying alternative methods for dealing with large scale jobs, this will include an improvement in ram usage by writing back the storage object every a given amount of results (this will provoke a rising down of the performances, but it will improve scalability), otherwise a single db file could be written for each blast result; in this case scalability will improve but managing of the results file could become more confusing.

Minor efforts will be devoted to *manage.py* module since we suppose users can modify methods to tailor them to their necessity.

Contact us

Massimiliano Orsini : orsini@crs4.it
Simone Carcangiu : simone.carcangiu@gmail.com

Terms of use

BlaSTorage has been developed by CRS4 Bioinformatics-Functional Genomics Unit, POLARIS scientific park, 09010 Pula, Italy. All rights to the BlaSTorage package are reserved to
CRS4 Bioinformatics, POLARIS scientific park, 09010 Pula, Italy.

BlaSTorage is a free and open source software toolkit, and distributed in the hope that it will be useful. In no event will CRS4 be liable to you for damage, including any general, special, consequential or incidental damage arising out of the use, modification or inability to use the program (including but not limited to loss of data or data being rendered inaccurate or losses sustained by you or third parties or a failure of the program to operate with any other programs). BlaSTorage can be used, redistributed and/or modified freely for non-commercial purposes subject to the original source is properly cited.

THIS PACKAGE IS PROVIDED “AS IS” AND WITHOUT WARRANTY OF ANY KIND, EITHER EXPRESSED OR IMPLIED, INCLUDING, BUT NOT LIMITED TO, THE IMPLIED WARRANTIES OF MERCHANTIBILITY AND FITNESS FOR A PARTICULAR PURPOSE.

Use of this package is taken as an agreement to these terms of usage.
